# Reliability and Validity of Key Performance Metrics of Modified 505 Test

**DOI:** 10.3390/life15020198

**Published:** 2025-01-28

**Authors:** Aleksandar Živković, Srdjan Marković, Ivan Cuk, Olivera M. Knežević, Dragan M. Mirkov

**Affiliations:** 1 Faculty of Physical Education and Sports Management, Singidunum University, 11000 Belgrade, Serbia; aleksandar.zivkovic@singidunum.ac.rs (A.Ž.); smarkovic@singidunum.ac.rs (S.M.); 2 Faculty of Sport and Physical Education, University of Belgrade, 11000 Belgrade, Serbia; olivera.knezevic@fsfv.bg.ac.rs (O.M.K.); dragan.mirkov@fsfv.bg.ac.rs (D.M.M.)

**Keywords:** sprint, COD, symmetry, reaction time, protocol

## Abstract

This study aims to explore the reliability and validity of essential performance metrics derived from a modified 505 Change of Direction (CoD) test. Twenty-seven physically active male participants (age: 18.77 ± 1.73 years) who engaged in three to five training sessions per week were assessed using both standard and modified sprint and CoD protocols. The evaluation focused on sprint time, CoD time, total time, and the Limb Symmetry Index (LSI). The modified protocols demonstrated good reliability for sprint and CoD times (ICC > 0.8, CV < 3%) and high validity, with strong correlations between standard and modified tests for sprint (r = 0.59–0.80) and CoD times (r = 0.58–0.80). The total time metric showed excellent reliability (ICC > 0.9), supporting its utility as a comprehensive measure. Reaction-time inclusion increased variability (CV > 7%) but enhanced ecological validity by reflecting real-world conditions. However, the LSI exhibited lower reliability (ICC < 0.6), indicating the need for further refinement. Combining multiple performance measures, the modified protocols streamlined assessments, reducing fatigue and redundancy without compromising precision. These findings highlight the potential of integrating multidimensional agility metrics in athletic evaluations, bridging the gap between controlled testing and competitive demands. Future research should explore broader populations and sport-specific stimuli to enhance applicability.

## 1. Introduction

Starting acceleration is critical to an athlete’s success in team sports like soccer, basketball, and handball [1,2,3]. Explosive bursts of speed are often decisive in high-pressure moments, whether gaining a positional advantage, intercepting a pass, or outmaneuvering an opponent. An accurate assessment of this ability is essential for identifying individual strengths and weaknesses, crafting effective training programs, and monitoring the impact of performance-enhancing interventions [4,5]. However, in team sports, athletes rarely accelerate in straight lines. Most movements involve accelerating, decelerating, turning, and decision making [6]. Combining sprint and turnaround tests enhances the ecological validity of the assessment (e.g., sprinting alone may not capture the complexity of change of direction (COD) in sports, while turnaround tests might overlook the significance of raw speed).

Traditionally, acceleration is measured using linear sprint tests, such as the 10 m sprint, while change of direction (CoD) ability is assessed separately. Testing CoD in team sports provides insight into an athlete’s readiness for frequent and rapid direction changes to evade opponents, maintain possession, or intercept the ball. CoD is often assessed through tests like the 505 CoD test, which evaluates an athlete’s capacity to decelerate, pivot, and reaccelerate, offering valuable insights into agility and balance [6,7,8,9,10]. While both methods are widely used and validated, they are typically performed independently, requiring multiple protocols to assess acceleration and CoD separately.

Recent studies have proposed combining these assessments to streamline testing and reduce redundancy. Metrics such as “total time” offer a comprehensive measure of acceleration and CoD performance, presenting an efficient alternative to traditional methods [6,11]. However, little research has compared results from combined protocols with those from separate assessments, raising important questions, such as whether combining the tests introduces fatigue-related biases or mask asymmetries between limbs. The Limb Symmetry Index (LSI), which quantifies differences in pivoting performance between dominant and non-dominant legs, may also vary depending on the testing protocol [8,12,13,14,15].

Integrating a 10 m sprint into the 505 CoD test offers the potential to streamline assessments and save time by consolidating multiple evaluations into one test. This would enable the measurement of additional metrics such as total time, Limb Symmetry Index (LSI), and CoD deficit. The CoD deficit isolates CoD ability from linear speed, providing a more refined evaluation of an athlete’s performance [6]. Adding a reaction-time element, such as initiating movement in response to a visual or sport-specific stimulus, further expands the scope of the test to include perceptual and decision-making components, which are critical in game-like scenarios [16]. This approach aligns with Sheppard and Young’s [17] definition of agility, emphasizing both physical and cognitive components, and addresses the limitations of traditional CoD tests that measure only pre-planned movements.

In particular, in sports such as basketball, handball or football, athletes must quickly perceive and interpret visual cues (e.g., the movement of opponents or the trajectory of the ball) and respond adequately. Therefore, training or testing protocols that include reaction-time tasks can enhance the ecological validity of assessments, making them more relevant to real-world performance [18]. While promising, these modifications require thorough validation to confirm their reliability and practical utility.

This study addresses these gaps by systematically evaluating the reliability and validity of a modified 505 CoD test that integrates a 10 m sprint. The key objectives were as follows: (1) to assess the reliability of key performance metrics, including sprint time, 505 CoD time, total time, and the LSI; (2) to examine the stability of the LSI across trials as an indicator of asymmetry; and (3) to evaluate the potential of reaction-time integration as a measure of multidimensional agility.

This research aims to provide actionable insights for enhancing performance evaluations in team sports by directly comparing standard and modified protocols.

## 2. Materials and Methods

### 2.1. Participants

This study involved twenty-seven male participants (age: 18.77 ± 1.73 years; body height: 182.8 ± 7.7 cm; body mass: 79.5 ± 10.3 kg; fat percentage: 14.1 ± 3.6%; mean ± SD). All participants were physically active sub-elite-level handball, basketball, and football players. They were engaged in training sessions 3 to 5 times per week and reported no neurological disorders or lower limb injuries in the past six months. Ethical approval was obtained from the ethical board of the Faculty of Sport and Physical Education, University of Belgrade (number 02-3182/24-2, date 13 November 2024.). Participants were thoroughly briefed about the testing procedures and provided written informed consent in accordance with the Declaration of Helsinki. For minors, consent was obtained from their parents or guardians. The privacy of the subjects’ data was ensured at all times.

### 2.2. Study Design

This study used a within-subject repeated-measures design to compare the outcomes of a standard testing protocol with two modified 505 CoD protocols. Testing was conducted across three days with a 48 h break between each session to minimize fatigue and ensure recovery.

- Day 1: Participants completed measurements of morphological characteristics (body mass, height, and body fat percentage) followed by the 10 m sprint test and the classic 505 CoD test. For the 505 CoD test, participants turned around both the dominant and non-dominant legs. The dominant leg’s superior motor control, force production, and coordination align with the demands of kicking mechanics. Therefore, the dominant leg was defined as the one used to kick a ball [19], with 21 participants reporting their right leg as dominant and six reporting their left. Participants were divided into two groups: one performed tests turning around the dominant leg first, and the other started with the non-dominant leg.
- Day 2: Participants performed the modified 505 CoD test, which integrated the 10 m sprint as an additional performance measure.
- Day 3: Participants performed the modified 505 CoD test with a visual signal to initiate movement, adding a cognitive component to the assessment.

For all sessions, the group composition and testing order remained consistent. Testing was conducted in the morning, with the same experienced measurer administering all assessments. Each session began with a standardized warm-up of 10–15 min, including running drills (ABCs) and calisthenics. Before each test, the procedures were explained in detail, and a demonstration was provided.

The testing was conducted towards the end of the season when researchers could control training activities and manage the workload of the subjects.

### 2.3. Procedures

#### 2.3.1. 10-m Sprint (S10m)

Two photocells (Running&Jumping All4Gym, Belgrade, Serbia) were positioned at the start and 10 m from the starting line. Participants were instructed to sprint as fast as possible across the 10 m distance without slowing down. The standing start method was used, and timing began when the infrared beam of the photocell at the starting line was broken. Each participant completed two trials with a 1 min pause between trials—enough to replenish more than 90% of the phosphocreatine stores [20,21]. Both trials were used for reliability analysis, and the better trial was used for validity calculations.

#### 2.3.2. Classic 505 CoD Test (C505)

**Figure 1:**
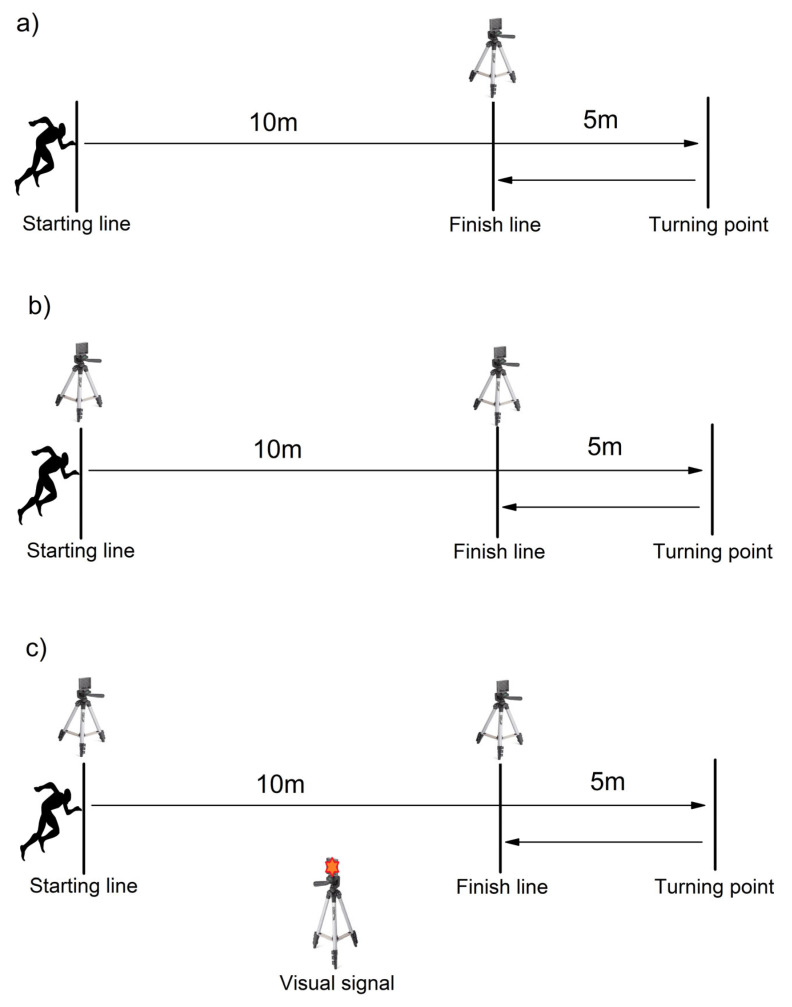
Graphic depiction of (**a**) classic 505 CoD test (C505), (**b**) modified 505 CoD Test (M505), and (**c**) modified 505 CoD Test with visual signal (RT505).

One photocell (Running&Jumping All4Gym, Belgrade, Serbia) was positioned 10 m from the starting line and 5 m before the turning point [22]. Participants sprinted 10 m, ran 5 m at maximum speed, pivoted 180 degrees, and reaccelerated 5 m back to the finish line (Figure 1a). Timing began when the photocell at the 10 m line was triggered and recorded the time for the 2 × 5 m section. Each participant performed four trials (two for each leg) with a 1 min rest between trials. The LSI was calculated as the ratio of the time recorded when pivoting around the non-dominant leg to the time recorded when pivoting around the dominant leg. All trials were used for reliability analysis, and the best trial was used for validity calculations.

#### 2.3.3. Modified 505 CoD Test (M505)

Two photocells (Running&Jumping All4Gym, Belgrade, Serbia) were positioned at the starting line and 10 m from the starting line. Participants sprinted 10 m, ran 5 m to the pivot point, pivoted 180 degrees, and reaccelerated 5 m back to the finish line (Figure 1b). Timing began when the infrared beam at the starting line was broken. Separate times were recorded for the 10 m sprint (M10m) and the 2 × 5 m segment (M505). The LSI was calculated similarly to the classic 505 test. Each participant performed six trials (three for each leg) with a 1 min rest between trials. All trials were used for reliability analysis, and the best trial was used for validity calculations.

#### 2.3.4. Modified 505 CoD Test with Visual Signal (RT505)

The setup was identical to the modified 505 test. However, a visual signal was used to initiate the sprint. A light positioned 5 m ahead at eye level served as the stimulus (Figure 1c), and timing began when the participant reacted to the signal and broke the infrared beam at the starting line. Separate times were recorded for the 10 m sprint (RT10m) and the 2 × 5 m segment (RT505). The LSI was calculated similarly. Each participant performed six trials (three for each leg) with a 1 min rest between trials. All trials were used for reliability analysis, and the best trial was used for validity calculations.

### 2.4. Statistical Analysis

Descriptive statistics, including the mean and standard deviation, were calculated prior to all statistical tests. The Kolmogorov–Smirnov test, alongside visual inspections of histograms and QQ plots, confirmed the normality of data distribution.

To evaluate the reliability of the classic and modified 505 test variables, the standard error of measurement (SEM), the coefficient of variation (CV), and intraclass correlation coefficients (ICCs) with 95% confidence intervals were computed. ICC values were interpreted as follows: <0.5 (poor reliability), 0.5–0.75 (moderate reliability), 0.75–0.9 (good reliability), and >0.9 (excellent reliability) [23]. Paired sample *t*-tests and repeated one-way ANOVA were conducted to assess differences between trials.

The Pearson correlation coefficient was used to evaluate the concurrent validity of the classic and modified 505 test variables. Correlation magnitudes were classified as small (r = 0.10–0.29), moderate (r = 0.30–0.49), and large (r = 0.50–1.0) [24]. Following repeated one-way ANOVA, Bonferroni post hoc analyses were performed to explore significant differences further.

Cohen’s d effect size was calculated to assess the practical significance of differences, with thresholds defined as trivial (<0.2), small (0.2–0.5), moderate (0.5–0.8), large (0.8–1.3), and very large (>1.3) [24]. Effect sizes were computed using the harmonic mean of the standard deviations (SDs) of the compared conditions, with a threshold of d = 0.20 considered the minimum for practical significance. Effects were deemed unclear if the 95% confidence intervals (CIs) included both positive and negative values; otherwise, effects were classified as clear.

The level of statistical significance was set at *p* < 0.05. All statistical analyses were performed using Microsoft Office Excel 2017 (Microsoft Corporation, Redmond, WA, USA) and SPSS 26 (IBM Corporation, Armonk, NY, USA).

## 3. Results

### 3.1. Reliability

Table 1 details the reliability results, including changes in mean values, absolute and relative reliability, and systematic differences across trials. These results cover the standard 10 m sprint and 505 CoD test, as well as two modified versions of the 505 test that incorporate the 10 m sprint timing and reaction time.

#### 3.1.1. 10 m Sprint Times

The 10 m sprint demonstrated excellent reliability across all testing protocols. The standard test achieved an ICC of 0.845 and a CV% of 2.48%, indicating strong consistency. The modified protocols maintained similarly high reliability, with CV% values ranging from 1.80% to 2.23% and ICC values between 0.868 and 0.933. Changes in mean values were minor, ranging from −1.64% in the standard test to 1.66% in the modified protocols. No significant differences between trials were observed in most cases.

#### 3.1.2. 505 CoD Times

The 505 CoD test exhibited moderate to excellent reliability across all conditions, depending on the protocol and the leg assessed. For the standard test, ICC values were 0.704 for the non-dominant leg and 0.744 for the dominant leg, with CV% values ranging from 2.84% to 3.11%. The modified protocols demonstrated reliability metrics consistent with the standard test, with ICC values ranging from 0.839 to 0.886 and CV% values between 1.82% and 2.13%. Changes in mean values were minimal (under 1.3%), and no significant differences between trials were observed.

#### 3.1.3. Reaction Times

Adding a reaction-time component in the modified protocols introduced slightly higher variability than time-based metrics. Reaction times showed CV% values of 8.64% for the non-dominant leg and 7.48% for the dominant leg, with ICC values of 0.799 and 0.805, respectively, indicating good relative reliability. Changes in mean values ranged from −2.91% to 1.90%, and no significant differences between trials were detected.

#### 3.1.4. Limb Symmetry Index (LSI)

The LSI exhibited low ICC values across all protocols (0.212–0.566), indicating poor relative reliability. However, the CV% ranged between 2.51% and 3.29%, suggesting a stable and narrow range of values. Mean values remained consistent (approximately 0%), indicating that the protocol modifications did not affect symmetry measurements. Significant differences between trials were observed only in the standard test (*t*-test = −2.391), suggesting a potential sensitivity of the LSI to testing conditions.

#### 3.1.5. Total Time (TT)

Total time remained a highly reliable metric across all protocols, with ICC values exceeding 0.90 and CV% values below 2%. Changes in mean values were minimal (<2%). However, significant differences between trials were observed in the modified protocols with the reaction time (*t*-test = 4.719–9.097), reflecting sensitivity to testing conditions rather than a reduction in reliability.

### 3.2. Validity

**Figure 2:**
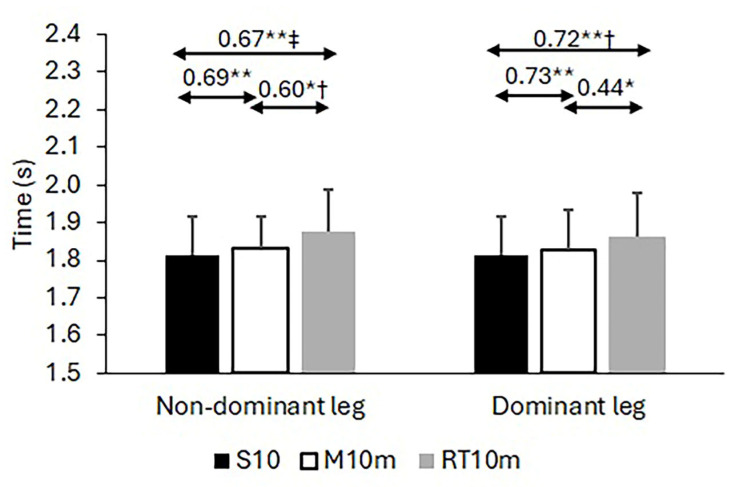
Validity of 10 m sprint times obtained from standard 10 m sprint and classic version of 505 test. Note: * significant correlation at *p* < 0.05; ** significant correlation at *p* < 0.01; †—difference significant at *p* < 0.05; and ‡—difference significant at *p* < 0.01.

**Figure 3:**
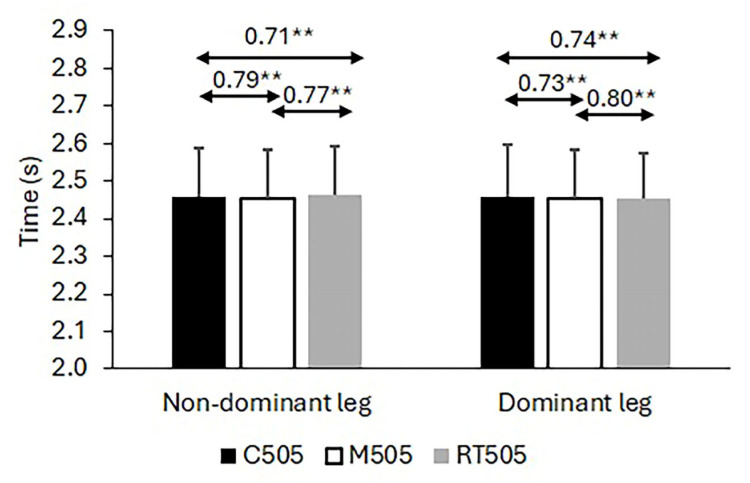
Descriptive statistics and correlation coefficients for times recorded in standard (505C) and two modified 505 tests (M505 and RT505). Note: ** significant correlation at *p* < 0.01.

**Figure 4:**
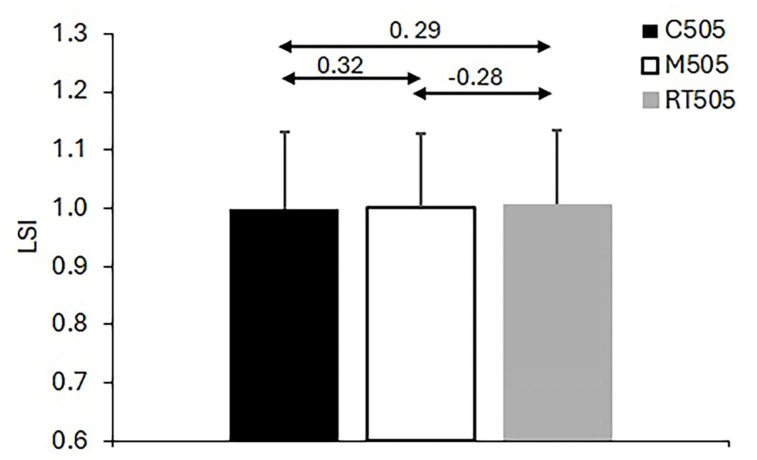
Descriptive statistics and correlation coefficients for the Limb Symmetry Index calculated between the non-dominant and dominant leg times recorded using three versions of 505 tests.

Regarding the calculated Pearson correlation coefficients among the 10 m sprint times and times on the 505 CoD tests, the results demonstrate significant associations for both the 10 m sprint times and the 505 test times across the standard and modified protocols (see Figure 2). For the 10 m sprint, the correlation coefficients between the standard test and the corresponding 10 m sprint times obtained within the modified 505 protocols ranged from r = 0.592 to r = 0.801, all significant at *p* < 0.01 (coefficients and significance levels are annotated in Figure 2). Similarly, for the 505 CoD test times, strong correlations were observed between the classic and modified protocols, with coefficients ranging from r = 0581 to r = 0.801, all significant at *p* < 0.01 (annotated in Figure 3). In contrast, the correlations for the LSI, computed as the ratio of non-dominant to dominant leg times, were not significant (r = −0.28 to 0.32, all *p* > 0.10) and notably negative between the two modified versions of the 505 test. These results and the observed variability in LSI values are presented in Figure 4.

Regarding the results of the repeated-measures ANOVA, the main effect of the factor Test was significant for 10 m sprint times and 505 CoD times with the dominant pivoting leg. In comparison, no significant effect was observed for the 505 CoD times with the non-dominant leg.

#### 3.2.1. 10 m Sprint Times

A significant main effect of the Test was observed for 10 m sprint times across all conditions (F2, 52 = 5.556, *p* = 0.007). Post hoc comparisons showed that the standard 10 m sprint was not significantly different from the first modified protocol (*p* = 1.000, d = −0.09, 95% CI [−0.20, 0.02]) but was significantly faster than the second modified protocol (*p* = 0.005, d = −0.46, 95% CI [−0.57, −0.35]). The two modified protocols were also significantly different (*p* = 0.035, d = −0.44, 95% CI [−0.55, −0.33]). Pairwise comparisons are annotated in Figure 2.

#### 3.2.2. 505 CoD Times

A significant main effect of the Test was observed for 505 CoD times with the dominant pivoting leg (F2, 52 = 4.930, *p* = 0.011) but not with the non-dominant leg (F2, 52 = 1.369, *p* = 0.263). For the dominant leg, post hoc comparisons revealed no significant difference between the standard and first modified protocols (*p* = 0.867) or between the first and second modified protocols (*p* = 1.000), but the standard test was significantly slower than the second modified protocol (*p* = 0.011). Results are annotated in the corresponding figures.

#### 3.2.3. LSI

No significant main effect of the Test was found for the Limb Symmetry Index (F2, 52 = 0.495, *p* = 0.610). These results emphasize consistent LSI values across test conditions, as shown in Figure 4.

## 4. Discussion

This study aimed to validate the reliability of a modified 505 CoD test that integrates a 10 m sprint while evaluating the utility of the total time (TT) and the Limb Symmetry Index (LSI) as novel metrics. Additionally, reaction time was incorporated as a perceptual-motor element to create a more multidimensional assessment of agility. These modifications were designed to improve the efficiency and comprehensiveness of athletic testing protocols.

The findings demonstrated that the modified protocols maintained reliability comparable to the standard tests. Sprint and CoD times were consistent across trials for both dominant and non-dominant legs, supporting the feasibility of consolidating these metrics into a single protocol. This reduces the need for multiple assessments, saves time, and minimizes fatigue while preserving diagnostic accuracy. Furthermore, this “breakdown of metrics” enables coaches to adapt training programs to an athlete’s specific needs. For example, if the 10 m time is slow, the athlete should focus on acceleration drills, while, if the 10 m is fast but the total test time is slow, the athlete should focus on CoD techniques or core stability exercises.

The TT metric proved to be a reliable composite measure, capturing both acceleration and CoD performance in a single outcome. This aligns with previous studies [6,25], highlighting the practicality of integrated metrics for use in high-intensity sports environments. TT’s reliability makes it a valuable tool for simplifying performance evaluations without compromising precision.

The addition of reaction time to the RT505 test introduced a cognitive element, addressing an important aspect of game-like scenarios. Athletes demonstrated slower sprint times in the RT505 compared to standard protocols, reflecting the additional perceptual-motor demands of responding to a visual stimulus. While this trade-off slightly increased variability, it enhances the ecological validity of the test by simulating real-world conditions where athletes must react to external cues. These results correspond with earlier studies that highlight the role of perceptual skills in reactive agility [26,27].

The LSI metric showed lower reliability across all protocols, likely due to variability in pivoting tasks. However, the narrow range of values (0.99–1.01) indicates potential for detecting subtle asymmetries. This metric remains important for identifying imbalances that could influence performance or injury risk [13,28,29,30]. Future studies could explore alternative approaches to improve its consistency, particularly in the context of injury prevention and rehabilitation.

The modified 505 COD test and RT505 provide a comprehensive solution for assessing physical and cognitive performance dimensions. By integrating acceleration, COD, and reaction time into a unified protocol, these tests bridge the gap between controlled laboratory conditions and the unpredictable demands of real-world sports. This multidimensional approach supports more nuanced evaluations and better-informed training strategies, ultimately enhancing athlete development and performance monitoring. Furthermore, integrating a 10 m sprint into the modified test addresses key limitations of the standard 505 test, including its lack of cognitive engagement and potential to induce fatigue from repetitive isolated tests. Traditional agility assessments focus solely on physical attributes, often neglecting critical perceptual-motor components essential for real-world athletic performance [31]. The modified protocol reduces cumulative fatigue and testing time while facilitating the measurement of total time (TT) and the Limb Symmetry Index (LSI), metrics that broaden the scope of athletic capacity [16,32]. By incorporating cognitive demands, such as responding to visual or sport-specific stimuli, the tests align with Sheppard and Young’s [33] definition of agility, which emphasizes decision-making and reaction elements alongside physical speed. This holistic approach reliably distinguishes between athletes of varying skill levels, better replicates game-like scenarios, and provides a more comprehensive, multidimensional evaluation of athletic abilities [31,34].

Some limitations of this study should be acknowledged. Firstly, the sample consisted of physically active males, which may restrict generalizability to other populations, such as female athletes or elite performers. Secondly, the visual stimulus used in the RT505 test may not fully replicate the unpredictability of competitive environments. In particular, it represents the simple reaction time, while team sports often consist of multilevel choice reaction times [35]. Future research could incorporate more dynamic or sport-specific stimuli to enhance ecological validity. Finally, variability in pivoting tasks likely contributed to the LSI’s lower reliability, warranting refinement of this metric for broader application.

## 5. Conclusions

This study offers important insights into the assessment of acceleration, agility, and change of direction (CoD) in athletic performance, particularly for team sports. By integrating a 10 m sprint into the 505 CoD test, we developed a more efficient and consolidated protocol that combines multiple performance measures into a single assessment. The findings support the utility of this modified test in evaluating starting acceleration and CoD performance without compromising reliability. The addition of a reaction-time component further expands the scope of traditional evaluations by addressing both physical and cognitive dimensions of agility. This enables a more holistic understanding of an athlete’s performance, particularly in game-like scenarios requiring quick perceptual-motor responses. However, when the primary focus is on starting acceleration, the modified 505 CoD test without reaction-time integration is recommended, as it efficiently measures acceleration and CoD performance while providing a reliable composite result (total time).

In contrast, where the interaction between reaction time and starting acceleration is of interest, combining the RT505 test with a separate 10 m sprint without external stimuli can yield valuable insights. Such an approach allows for a direct comparison between reaction-based and standard sprint conditions, helping assess the cognitive load’s impact on athletic performance. Although the LSI exhibited lower reliability in this study, it remains a valuable metric for identifying asymmetries that may inform injury prevention and rehabilitation strategies.

By addressing both physical and perceptual-motor aspects of athletic performance, the modified 505 CoD test and RT505 protocol represent significant advancements in performance evaluation. These innovations bridge the gap between controlled laboratory settings and the dynamic, unpredictable nature of real-world sports. The streamlined and comprehensive nature of these tests provides athletes, coaches, and sports scientists with effective tools for assessing performance, optimizing training programs, and monitoring athletic development.

## Figures and Tables

**Table 1:** Reliability data for standard and modified protocols (mean ± SD across trials, change in mean, CVs, standard errors, ICCs and differences between trials).

Test	Variable	Trial 1	Trial 2	Trial 3	2-1%	3-2%	CV%	SEM	**ICC (95% CI)**	**Ttest/ANOVA**
**Standard 10 m sprint and 505 test**	10 m	1.849 ± 0.117	1.822 ± 0.108	-	−1.64	-	2.48	0.05	0.845 (0.719, 0.917)	1.884
	505N	2.484 ± 0.143	2.513 ± 0.129	-	1.27	-	3.11	0.08	0.704 (0.482, 0.837)	−1.408
	505D	2.516 ± 0.132	2.486 ± 0.132	-	−1.19	-	2.84	0.07	0.744 (0.558, 0.858)	1.598
	LSI 505	0.989 ± 0.033	1.012 ± 0.040	-	2.32	-	3.29	0.03	0.212 (−0.161, 0.513)	−2.391 *
**505 modified**	10mN	1.835 ± 0.104	1.858 ± 0.085	1.857 ± 0.084	1.38	−0.10	1.83	0.03	0.868 (0.774, 0.927)	1.767
	10mD	1.828 ± 0.136	1.869 ± 0.099	1.857 ± 0.106	1.66	−0.57	2.23	0.04	0.868 (0.773, 0.927)	2.436
	505N	2.475 ± 0.125	2.499 ± 0.123	2.481 ± 0.131	1.02	−0.22	2.13	0.05	0.839 (0.731, 0.910)	1.192
	505D	2.468 ± 0.141	2.492 ± 0.116	2.478 ± 0.108	1.11	−0.53	1.82	0.04	0.876 (0.789, 0.931)	2.121
	LSI 505	1.002 ± 0.035	1.005 ± 0.036	1.003 ± 0.04	0.25	−0.05	2.52	0.02	0.566 (0.339, 0.740)	0.237
	TTN	4.274 ± 0.225	4.364 ± 0.193	4.326 ± 0.194	2.22	−0.27	1.51	0.06	0.908 (0.841, 0.950)	9.097 *
	TTD	4.210 ± 0.194	4.354 ± 0.206	4.335 ± 0.199	2.04	−0.40	1.55	0.07	0.901 (0.829, 0.946)	9.055 *
**505 modified RT**	RTN	0.613 ± 0.098	0.596 ± 0.110	0.604 ± 0.093	−2.91	1.90	8.64	0.05	0.799 (0.669, 0.886)	0.710
	RTD	0.615 ± 0.068	0.619 ± 0.112	0.605 ± 0.106	−0.28	−3.88	7.48	0.04	0.805 (0.679, 0.890)	1.790
	10mN	1.879 ± 0.106	1.903 ± 0.097	1.883 ± 0.101	1.09	−0.57	1.80	0.03	0.904 (0.834, 0.947)	1.789
	10mD	1.893 ± 0.117	1.878 ± 0.134	1.883 ± 0.113	−0.84	0.95	1.82	0.03	0.933 (0.885, 0.964)	1.605
	505N	2.484 ± 0.151	2.489 ± 0.125	2.493 ± 0.126	−0.04	−0.19	1.91	0.05	0.886 (0.805, 0.937)	0.054
	505D	2.491 ± 0.106	2.472 ± 0.102	2.479 ± 0.128	−0.29	−0.11	1.88	0.05	0.841 (0.732, 0.911)	0.268
	LSI 505	1.003 ± 0.038	1.005 ± 0.031	1.003 ± 0.028	−0.04	−0.59	2.51	0.02	0.432 (0.163, 0.650)	0.287
	TTN	4.355 ± 0.228	4.403 ± 0.205	4.404 ± 0.249	1.35	−0.08	1.65	0.07	0.904 (0.834, 0.948)	4.719 *
	TTD	4.381 ± 0.206	4.348 ± 0.217	4.374 ± 0.222	−0.78	0.60	1.60	0.07	0.905 (0.839, 0.947)	1.325

Note: S—10 m sprint; C505—classic 505 test; M-—modified 505 test; RT-—modified 505 with reaction time; 10 m—ten meter distance; -N—nondominant leg; -D—dominant leg, LSI 505—Leg Symmetry Index; RT—reaction time; TT—total time; and * difference significant at *p* < 0.05.

## Data Availability

The datasets presented in this article are not readily available because the data are part of an ongoing study. Requests to access the datasets should be directed to corresponding authors.
